# Formulation of Biologically-Inspired Silk-Based Drug Carriers for Pulmonary Delivery Targeted for Lung Cancer

**DOI:** 10.1038/srep11878

**Published:** 2015-08-03

**Authors:** Sally Yunsun Kim, Deboki Naskar, Subhas C. Kundu, David P. Bishop, Philip A. Doble, Alan V. Boddy, Hak-Kim Chan, Ivan B. Wall, Wojciech Chrzanowski

**Affiliations:** 1 Faculty of Pharmacy, The University of Sydney, Sydney, NSW, 2006, Australia; 2 Department of Biochemical Engineering, University College London, London WC1E7JE, United Kingdom; 3 Department of Biotechnology, Indian Institute of Technology Kharagpur, West Bengal - 721302, India; 4 Elemental Bio-imaging Facility, University of Technology Sydney, Broadway, NSW, 2007, Australia; 5 Department of Nanobiomedical Science & BK21 Plus NBM Global Research Center of Regenerative Medicine, Dankook University, Cheonan 330-714, Republic of Korea

## Abstract

The benefits of using silk fibroin, a major protein in silk, are widely established in many biomedical applications including tissue regeneration, bioactive coating and *in vitro* tissue models. The properties of silk such as biocompatibility and controlled degradation are utilized in this study to formulate for the first time as carriers for pulmonary drug delivery. Silk fibroin particles are spray dried or spray-freeze-dried to enable the delivery to the airways *via* dry powder inhalers. The addition of excipients such as mannitol is optimized for both the stabilization of protein during the spray-freezing process as well as for efficient dispersion using an *in vitro* aerosolisation impactor. Cisplatin is incorporated into the silk-based formulations with or without cross-linking, which show different release profiles. The particles show high aerosolisation performance through the measurement of *in vitro* lung deposition, which is at the level of commercially available dry powder inhalers. The silk-based particles are shown to be cytocompatible with A549 human lung epithelial cell line. The cytotoxicity of cisplatin is demonstrated to be enhanced when delivered using the cross-linked silk-based particles. These novel inhalable silk-based drug carriers have the potential to be used as anti-cancer drug delivery systems targeted for the lungs.

Silk has been used for centuries as fabric as well as in cosmeceutics. It is recently attracting immense attention in biomedical applications^1^. Silk fibroin, a major protein in silk cocoon is used as surgical sutures since decades ago and is known for properties including controllable biodegradability, superior mechanical strength and stability, elasticity and biocompatibility^1^. Silk fibroin is widely established as a platform for the controlled delivery of various drugs via formulations of microparticles, spheres, films, coatings, and hydrogels^1^,^2^.

Silk fibroin-based formulations are valuable not only due to their non-toxic and non-antigenic nature, but also for their stability during storage as well as *in vivo*^3^. Silk fibroin can be processed in an aqueous environment and are capable of being degraded into amino acids that are well absorbed by the body^4^. Silk-based drug delivery is significant because, unlike other polymer biomaterials, they can be processed in aqueous systems in mild conditions of temperature, pressure and neutral pH without the need for harsh manufacturing conditions, which may damage the incorporated drug^5^,^6^,^7^. Further extended applications of silk fibroin for drug delivery include delivery of growth factors for tissue repair, as well as delivery of medications for epilepsy^1^,^2^.

A few recent studies highlight the advantages of combining silk fibroin with other bioactive materials such as albumin, chitosan, hyaluronic acid, polyprolactone and aloe vera in drug delivery and tissue engineering applications^8^,^9^,^10^,^11^. The most common reason for the incorporation of silk fibroin was to improve the mechanical properties upon incorporation into three-dimensional scaffolds. Other specific reasons included enhancement of cell attachment^11^, improved drug release kinetics^8^,^9^,^10^ and superior blood compatibility^9^. Importantly, the hemocompatibility observed for chitosan/silk-based scaffolds was found to be associated solely with the presence of silk fibroin in these scaffolds^9^.

Despite the broad use of silk in biomedical applications, its potential as drug carriers for targeted delivery to the lungs is not investigated and silk-based delivery systems are yet to be developed into an inhalable form. Therefore, this study aims to develop novel, biologically-inspired silk-based inhalable drug delivery systems that target the lungs. Targeted delivery of drugs to the lungs for the treatment of chronic lung infections, lung cancers, tuberculosis and other respiratory conditions is emerging as an innovative area of research over the last decade^12^,^13^. Potential advantages of targeted delivery include the reduction of dosage and side effects of the drug compared to systemic drug delivery^13^. The majority of commercially available inhalation products are for local treatment of lung diseases. A number of inhalation products designed to treat systemic diseases are undergoing clinical development^14^. The lung is an attractive target for drug delivery due to the avoidance of first-pass metabolism, the large surface area and the rapid onset of action leading to enhanced absorption of drugs^13^,^15^,^16^.

Novel routes for the administration of cancer therapies are being investigated for decades^17^. Among the novel routes, pulmonary drug delivery for treatment for lung cancer is widely researched in the last two decades due to many obvious advantages including reduction of dosage required and subsequently reduced systemic side effects^17^. Cisplatin, a platinum-based first-line chemotherapy drug for lung cancer, is currently being delivered intravenously^18^. The conventional cisplatin formulation, when delivered intravenously, is widely distributed into the body fluids and tissues. This distributed drug rapidly binds to the tissue and plasma proteins leading to low bioavailability of the drug to the target organ^19^. The majority of the drug fails to reach the target site in the lungs. Instead, highest concentrations are found in the kidneys and are excreted from the body predominantly via renal excretion^19^. There are common systemic side effects such as nephrotoxicity, ototoxicity, mucositis and myelosuppression often leading to life-threatening septicaemia^19^. Furthermore, the cytotoxic effect of cisplatin in currently marketed formulations is short lasting and requires frequent dosing to maintain the concentration of the drug within the therapeutic window^19^,^20^. This increases the costs of treatment because a health care professional is required for the administration of every intravenous dose^19^.

A549 human lung epithelial cells are responsible for the diffusion of substances across the alveoli of the lungs. In this study, the cytocompatibility of silk and cytotoxicity of silk particles loaded with cisplatin are assessed. A previous study demonstrates the cytotoxic responses of A549 cells to cisplatin alone and compares with the combination with another anticancer drug imatinib using the MTT assay^21^. Kim *et al.* (2006) and Zhang *et al.* (2014) show the enhancement of chemosensitivity of A549 cells toward the cisplatin^22^,^23^. When micro-carriers are used to deliver cisplatin via inhalation, cisplatin is delivered preferentially to the lungs, and therefore leads to enhanced therapeutic efficacy and reduced side effects^24^.

In this study, we investigated whether the novel and biologically-inspired silk-based drug delivery systems are suitable for inhalation to achieve deposition in the lower airways. The *in vitro* experiments are designed to assess the compatibility of silk-based drug carriers with human lung epithelial cells. It is also interesting to know how the formulations of carriers can be engineered to enhance the cytotoxicity of cisplatin by increasing the extent of release from the carrier particles. The results indicate that this silk-based drug delivery system has the potential to be used in the targeted treatment of lung cancer.

## Materials and Methods

### Silk fibroin isolation and purification

Silk fibroin was extracted from mulberry silk cocoons of *Bombyx mori* (Bm) obtained from West Midnapore district of West Bengal, India as described previously with slight modifications^3^. Briefly the cocoons were cut into fine pieces and boiled with 0.02 M aqueous Na_2_CO_3_ solution for 30 min at 100 °C to remove the silk protein sericin. The degummed fibers were dissolved in 9.3 M aqueous LiBr solution and the resulting solution was dialyzed using 12 kDa molecular weight cut off dialysis membrane against deionized water for 2 days with frequent water change. The resulting silk fibroin solution was used to determine its concentration using the Bradford method. 2% protein solution was freeze dried to get the pure protein in dry form.

### Formulation of silk-based particles

Two methods were used to formulate silk-based particles: spray drying and spray-freeze-drying. Spray drying is one of the most common methods used to produce inhalable powders. Spray-freeze-drying is a method that produces higher yields of particles with high porosity, favorable for inhalation^25^. For both methods, the freeze dried silk fibroin was weighed and simply dissolved in deionized water to achieve Bm fibroin 2% (w/v) solution. The dissolution was carried out at 45 °C with stirring and sonication for 15 min. Any undissolved parts were removed by filtering through 0.22 μm syringe filters (Millipore, Millex^®^ GP Filter Unit, Millipore Express PES Membrane, Darmstadt, Germany).

#### Incorporation of cisplatin and cross-linking of silk fibroin

Cisplatin was incorporated at concentrations of 0.05% (w/v) into the silk formulations. In order to produce cross-linked silk formulations, genipin was added to the silk solutions at 0.05% (w/v) prior to the incorporation of cisplatin. For cross-linking, the silk aqueous solution was mixed using a magnetic stirrer for 15 hours at room temperature.

#### Fabrication of particles from spray-drying

Silk formulations were spray-dried using a lab scale spray-dryer (Büchi Mini Spray-Dryer B-290, BüchiLabortechnik, Flawil, Switzerland). An open loop was used with Büchi Dehumidifier B-296 in blowing mode. The operation conditions were liquid feed rate at 4.2 ml/min, aspiration at 100%, airflow with gas supply pressure 55 mbar, and inlet and outlet temperatures at 120 °C and 66–80 °C, respectively.

#### Fabrication of particles from spray-freeze-drying

For spray-freeze-drying silk solutions, trehalose or mannitol was incorporated at concentrations of 0.5% or 1% (w/v) as a lyoprotectant, to protect the structure of silk fibroin at the low temperatures during the drying process. The solutions were sprayed using an ultrasonic nozzle (Sonozap 130K50ST) powered by an ultrasonic generator (SonaerInc, New York, USA) with a constant flow rate of 0.5 ml/min controlled using a PHD 2000 syringe pump (Harvard Apparatus, Holliston, MA, USA). Particles were collected in liquid nitrogen and were dried in a freeze dryer (Christ Alpha 1–4, B. Braun Biotech International, Melsungen, Germany) under vacuum ranging from 0.07 to 0.12 mbar, at −20 °C for 24 hours of primary drying. This was followed by secondary drying at increasing temperatures of 10 °C per hour until the final temperature of 20 °C was reached.

### Characterization of silk-based particles for pulmonary delivery

#### Particle size distribution by dynamic light scattering

Geometric particle size and the size distribution of the particles were measured by laser diffraction using Mastersizer (Malvern Instruments, Worcestershire, UK). Dry dispersion with refractive index of 1.0 was used. Mass median diameter (D(0.5)), representing the diameter at which 50% of the particles by mass are larger and 50% are smaller, was considered as the average particle diameter by mass.

#### Particle size and morphology

Scanning electron microscopy (SEM) was used to observe the changes in morphology and size of the particles. Samples were sputter coated with approximately 20 nm thick gold using a K550X sputter coater (Quorum Emitech, Kent, UK). Images were obtained using Hitachi S-4500 field emission scanning electron microscope (Hitachinaka-shi, Ibaragi, Japan).

#### Analysis of surface topography and roughness

Atomic force microscopy (AFM) was used for observing particle size and imaging 3D surface topography. The particles were dispersed onto Tempfix^®^ (Plano GMBH, Wetzlar, Germany) prepared on a glass slide. MFP-3D-BIO^TM^ AFM (Asylum Research, Santa Barbara, USA) was used in AC mode using a silicon tip AC-160 (Olympus, Japan) with scan rate of 0.30–0.40 Hz. Surface roughness was measured and the average roughness (Ra, or average deviation) was compared between samples.

#### Determination of amorphous or crystalline structures

X-ray diffraction was used to determine whether the particles are amorphous or crystalline. An x-ray diffractometer (XRD-6000, Shimadzu Scientific Instruments, Tokyo, Japan) was used to measure diffraction pattern for each sample under ambient conditions with angular increments of 0.02° covering a 2θ range of 2 to 70°.

#### *In vitro* aerosolisation performance and lung deposition

In order to assess the *in vitro* aerosolisation performance, the aerodynamic diameters of the particles were calculated after dispersion of particles using a next generation impactor (NGI) (Copley Scientific Limited, Nottingham, United Kingdom). A 5 mg of sample inside a gelatin capsule (size 3) was dispersed using an Aeroliser^®^ (Pharmaxis Ltd, Frenchs Forest, Australia), at 60 L/min for 4 seconds. The particles were collected at 7 stages, each with aerodynamic diameter cut-off points 8.06, 4.46, 2.82, 1.66, 0.94, 0.55 and 0.34 μm when dispersed at 60 L/min^26^. The particles collected at each stage were dissolved using 5 ml of calcium chloride (CaCl_2_) 60% solution, selected as base medium due to its ability to solubilise silk fibroin. Then silk fibroin was quantified using a UV-Vis spectrophotometer with λ_max_ at 274 nm (UV-1800, Shimadzu Corporation, Tokyo, Japan). The proportion of particles with suitable aerodynamic diameters for inhalation (less than 5 μm) was determined using the standard curve generated.

### *In vitro* release of cisplatin

*In vitro* drug release studies were performed with a multi-station Franz cell station (VB6; PermeGear Inc, Hellertown, PA, USA). Franz cell method allows the mimicking of the air-liquid interface present in the lungs, and it is a widely accepted *in vitro* approach in comparing the drug release profiles of inhalation dry powder formulations^27^,^28^. The diffusion cells were stirred at a constant rate and thermoregulated with a water jacket maintained at 37 °C ± 0.5 °C *via* a circulating water bath. Phosphate-buffered saline (PBS) at pH 7.4 was used as the receptor fluid. 2 mg samples of cross-linked and non-cross-linked silk-cisplatin particles were evenly spread on top of 0.25 μm filter paper (Whatman^®^). At each pre-determined time point (20 min, 40 min, 1 h, 2 h, 4 h and 5 h), 2 ml sample was taken out for detection of cisplatin and 2 ml fresh PBS was added to maintain constant sink volume.

For the detection of cisplatin in each sample, inductively coupled plasma mass spectrometry (ICP-MS, Agilent Technologies 7500cx) was used with sample introduction *via* a micromist concentric nebuliser (Glass expansion) and a Scott type double pass spray chamber cooled to 2 °C. The ICP operating parameters and the lens conditions were selected to maximise the sensitivity of a 1% HNO_3_:HCl solution containing 1 ng/ml of Li, Co, Y, Ce and Tl. Calibration curves were constructed and the results were analysed using Agilent Technologies Masshunter software.

#### Reagents and samples

A Pt stock solution was obtained from Choice Analytical (Thornleigh, Australia). Baseline nitric acid (HNO_3_) and hydrochloric acid (HCl) was purchased from Seastar chemicals (Sidney, Canada). The samples were diluted 1:3 in a 1% HNO_3_:HCl solution before analysis. The calibration standards were matrix matched to the samples.

### *In vitro* cytocompatibility of silk proteins and cytotoxicity of silk-cisplatin particles

#### Cell culture and maintenance

Human lung carcinoma epithelial cell line A549 was used to investigate the cytocompatibility of silk fibroin and to examine the cytotoxicity of cisplatin released from silk-based particles. Cells were maintained using “normal media” consisting of Dulbecco’s Modified Eagle’s Medium (DMEM) with 4500 mg/L D-glucose, L-glutamine, containing 10% fetal bovine serum (FBS), with 1% penicillin-streptomycin (Penstrep^®^) (All purchased from Gibco, Life Technologies, USA, except FBS from Sera Laboratories, UK) at 37 °C in 95% air and 5% CO_2_ atmosphere. Medium was renewed every 2 to 3 days and cells were passaged using Trypsin/EDTA (Sigma-Aldrich Ltd, Dorset, UK) when monolayer was 80–85% confluent. All polystyrene plastic flasks and plates used were of tissue culture grade.

#### Preparation of particle-conditioned media using formulated particles

For the purpose of testing the cytocompatibility and cytotoxicity of the particles, particle-conditioned media was prepared by dissolving the particles in normal media at 1 mg/ml concentrations for 24 hours at 37 °C in the water bath. The formulations used were silk alone, silk-cisp (containing silk + mannitol + cisplatin at the ratio of 10:20:1), or silk-cisp (cross-linked; containing silk + mannitol + cisplatin + genipin at the ratio of 10:20:1:1) formulations. The solutions were filter-sterilised through a 0.22 μm syringe filter (Millex^®^ GP Filter Unit, Millipore Express PES Membrane, Millipore, MA, USA).

#### Cell viability and proliferation assay

Cells were seeded at 2000 cells/well on 96-well plates and were allowed to attach to the bottom of the wells. Then normal media was replaced with particle-conditioned media prepared as described above. At each predetermined time point (days 1, 3 and 7), particle-conditioned media was replaced by 100 μl of fresh normal media. After one hour incubation, 10 μl of CCK-8 reagent (Dojindo Molecular Technologies Inc, Tokyo, Japan) was added to each well. After two hours, the optical density (OD) of each well was measured using a microplate reader at 450 nm (Tecan Safire^2^, Tecan Group Ltd, Seestrasse, Switzerland).

#### Picogreen^®^ DNA quantification assay

Cells were seeded at 2000 cells/well on 96-well plates and were treated with particle-conditioned media as described above. At 24 and 72 hours, cells were washed with PBS, detached and re-suspended in sterile water for cell lysis and release of DNA through the freeze-thaw cycles, repeated three times. The double stranded DNA (dsDNA) content in cells was measured using a Picogreen^®^ dsDNA Quantitation Kit (Invitrogen Molecular Probes, NY, USA), according to the manufacturer’s protocol. Tris-ethylenediamine-tetraacetic acid (TE) buffer was used to dilute the cell suspension to a total volume of 500 μl, then Quant-iT^TM^Picogreen^®^ reagent (Invitrogen Molecular Probes, NY, USA) 0.5% in TE buffer was added to each sample. After incubation at room temperature in the dark for 5 min, the fluorescence was measured using Fluostar Optima plate reader (BMG Labtech, Ortenberg, Germany) at excitation 450 nm / emission 544 nm.

#### Assessment of cell morphology

To assess changes of the cell morphology in response to the silk-based particles, cells were seeded at 2.5 × 10^5^ cells/well on 24-well plates. After 24 hours, normal media was replaced with particle-conditioned media as prepared above. The changes in cell morphology in each treatment group were observed and the phase images were taken using a transmitted light microscope (EVOS xl, Advanced Microscopy Group, Life Technologies, NY, USA) with 10× and 20× magnifications at 24 and 48 hours.

#### Immunocytochemistry

The presence of tight junction proteins were of importance to be observed as they are functional proteins that act as a barrier in the role of protecting the epithelium^29^. Immunocytochemistry protocol from Abcam^®^ was used with some modifications^30^. Briefly, cells were fixed in paraformaldehyde 4% with 10 min incubation at room temperature, followed by washing twice with cold PBS. Cells were permeabilized in 0.1% Triton X-100 (BDH Laboratory Supplies, England) for 20 min, washed three times with PBS (5 min per wash) followed by blocking using bovine serum albumin (BSA) 1% for 30 min.

Incubation in primary antibody anti-beta catenin, clone 7F7.2; IgG (Millipore, MA, USA) (1:250) was overnight at 4 °C in the dark. After washing with PBS, cells were incubated with secondary antibody Alexa Fluor^®^ 555 (Life Technologies, NY, USA) (1:200) in PBS for one hour at room temperature in the dark. Counterstaining with conjugated F-actin (ActinGreen^TM^ 488, Life Technologies, NY, USA) and DAPI (4′,6-Diamidino-2-Phenylindole, Dilactate) nucleus staining followed for 30 min and 5 min, respectively. Cells were imaged using a fluorescent microscope at 20× magnification (Nikon Eclipse TE2000-U inverted microscope, Nikon Instruments Inc., NY, USA).

#### Cell metabolism

Cell metabolism was evaluated by measuring glucose and lactate levels in the media, representing energy consumption and by-product using the YSI 2700 Select Biochemistry Analyzer (YSI Incorporated, Yellow Springs, USA). Samples of media were collected after 1, 3 and 7 days of incubation with media containing silk alone, silk-cisp, or silk-cisp (cross-linked) formulations. L-lactate and D-glucose levels in media were measured in g/L and were expressed in a single graph.

#### Cell wound repopulation assay

This assay was conducted in order to measure the extent of migration or repopulation in a two-dimensional wound created on a monolayer in the presence of the sample formulations. Cells were seeded at 2.5 × 10^5^ cells/well on 24-well plates and were cultured in normal media until 90% confluent. Then cells were incubated in low serum media (containing 1% FBS) for 24 hours prior to scratching a wound on the midline of the culture well using a pipette tip (200 μl). Then the media was replaced by particle-conditioned media. Wound images were taken using a transmitted light microscope (EVOS xl, Advanced Microscopy Group, Life Technologies, NY, USA) with 10× magnification at 0, 24, and 48 hours. ImageJ software program was used for quantitative analysis, to measure and compare the area of wound at each time point.

#### Cell migration and invasion

Cell migration and invasion due to the presence of silk-based particle-conditioned media were analyzed using Transwell^®^ cell culture chambers (8 μm pore size, BD Biosciences, Oxford, UK). Cells were kept in low serum media (containing 1% FBS) for 24 hours, then were trypsinized and resuspended in low serum media and placed in the upper chamber of the Transwell^®^ insert (50,000 cells/well). Cells were allowed to attach for one hour then the media in the lower chamber was replaced with particle-conditioned media (containing 10% FBS) to observe suppressed attraction. Concentration gradient was established using low serum media (1%) in the upper chamber and high serum media (10%) in the lower chamber. The cells were incubated for 8 hours in a humidified atmosphere with 95% air and 5% CO_2_ at 37 °C.

Then cells were fixed with 4% paraformaldehyde, and were stained for 30 min using 2% crystal violet in 10% ethanol. The non-invasive cells in the upper chamber were removed by wiping with a cotton swab. The cells in the lower surface of the transwell insert that migrated through the pores were imaged using a transmitted light microscope (EVOS xl, Advanced Microscopy Group, Life Technologies, NY, USA) at 10× magnification and the number of migrated cells were counted using ImageJ software. Average number of migrated cells per image was graphed and analyzed.

### Statistical analyses

All data were produced in triplicates (*n* = 3) for reliability. Data was analyzed and presented as means ± standard deviation. The differences between the experimental and control groups were analyzed using one-way analysis of variance (ANOVA) test. A p-value less than 0.05 was reported as having a statistically significant difference.

## Results and Discussion

### Formulation and characterization of silk-based particles for pulmonary delivery

#### Assessment of particle size and morphology

**Figure 1:**
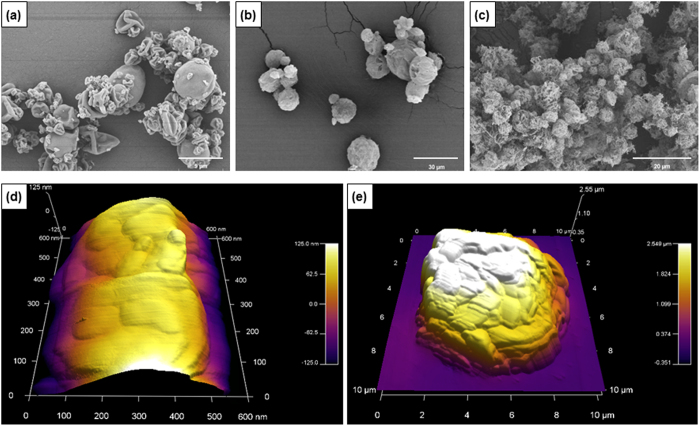
SEM(**a**–**c**) and AFM (**d**,**e**) micrographs of silk-based particles: (**a**) spray dried Bm fibroin; (**b**) spray-freeze-dried Bm fibroin 0.5% mannitol 1% cisplatin 0.1%; (**c**) spray-freeze-dried Bm fibroin 0.5% genipin 0.05% mannitol 1% cisplatin 0.05%, cross-linked for 15 hours; (**d**) spray dried Bm fibroin; (**e**) spray-freeze-dried Bm fibroin 2% trehalose 1%.

Particle sizes varied according to different parameters used on the spray dryer, and the optimization of inlet temperature, aspiration, atomization flow and the feed rate of solution led to inhalable sizes of silk particles. The lower feed rates of silk solutions into the spray dryer produced smaller particle sizes and higher atomization flow. As such, the smaller droplets of silk solution led to smaller particles. This was explained by more energy being supplied to break up the liquid droplet into smaller droplets during the atomization step^25^. After optimization of the parameters in order to produce inhalable particles, the sizes of spray-dried silk particles appeared more uniform and the majority of the particles were less than 5 μm (Fig. 1(a)), which is the desired size for pulmonary delivery^16^,^31^.

Laser diffraction for geometrical size analysis for spray dried particles consisting of Bm fibroin showed that mass median diameter (D(0.5)) / average particle diameter by mass, was 5.20 ± 0.69 μm. However, the particle size suitable for deposition in the lungs is determined by aerodynamic size instead of geometric diameters. The aerodynamic diameter (*d*_*a*_) for spherical particles is related to the particle density as well as the geometric diameter (*d*_*g*_), as shown in the following equation:


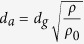


where *p* is the mass density of the particle and *p*_*0*_ is the unit standard particle density (1 g/cm^3^)^13^. Therefore, despite the spray-freeze-dried particles have larger geometric diameters, they can also have suitable aerodynamic sizes for potential successful deposition in the lower airways due to their porosity and low density^25^. The particles with aerodynamic diameters between 1 and 5 μm are capable of being deposited in the small airways and alveoli, while the particles with aerodynamic diameters between 5 and 10 μm are mainly deposited in the large airways^31^.

The collapsed particles observed on the SEM micrograph in Fig. 1(a) were the result of the spherical and hollow particles being unable to maintain their shape in the high vacuum used during the process of imaging. This confirmed that most spray-dried particles were hollow. The spray-freeze-dried particles were overall larger and more uniform in size compared to spray-dried particles as can be seen from the SEM images (Fig. 1(b,c)). The cross-linked silk-based particles were porous and aggregated (Fig. 1(c)). From observing the AFM images, the spray-dried particles appeared to have smoother surface than the spray-freeze-dried particles and the roughness expressed as Ra (average deviation) values measured were 26.52 nm and 420.81 nm respectively for spray-dried and spray-freeze-dried particles (Fig. 1(d,e)). The higher surface roughness on the spray-freeze-dried particles is a favourable characteristic for improved dispersion and enhanced aerosolisation efficiency^32^.

**Figure 2:**
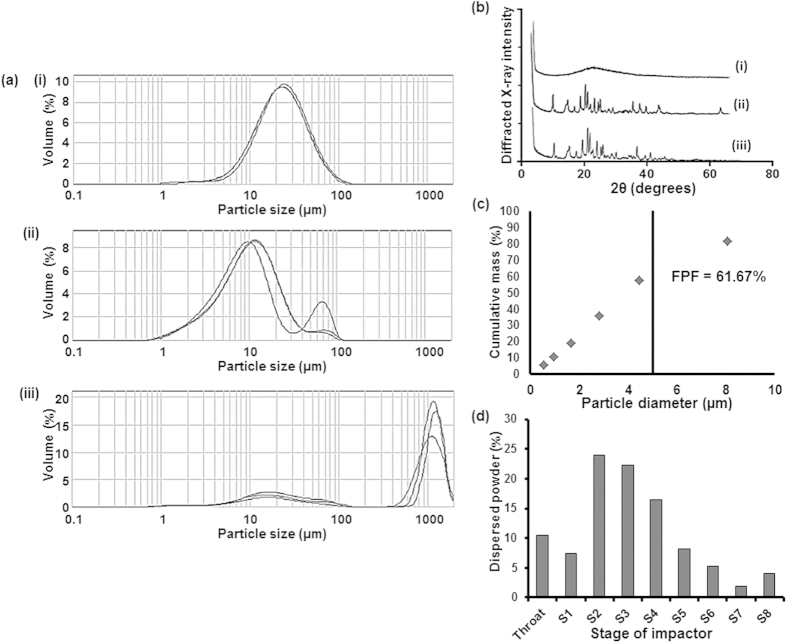
(**a**) Particle size distributions obtained from dynamic light scattering: (i) spray-freeze-dried silk + mannitol + cisplatin, (ii) spray-freeze-dried silk + mannitol + cisplatin + genipin, (iii) spray-freeze-dried silk + trehalose. (**b**) X-ray diffractograms of: (i) spray-dried silk alone, (ii) spray-freeze-dried silk + mannitol + cisplatin, (iii) spray-freeze-dried silk + mannitol + cisplatin + genipin, with angular increments of 0.02° covering a 2θ range of 2 to 70° show that particles are amorphous (smooth) or crystalline (with peaks). (**c**) Cumulative mass fraction for spray-dried Bm fibroin particles showing fine particle fraction (FPF) of particles less than 5 μm in diameter, obtained using *in vitro* lung deposition measurement. (**d**) The distribution of particles at airflow 60 L/min for 4 seconds using a next generation impactor; S1 to S7 represent stage numbers with aerodynamic diameter cut-off points 8.06, 4.46, 2.82, 1.66, 0.94, 0.55 and 0.34 μm respectively. S8 represents micro orifice collector, the last collection point on the impactor.

Porous polymeric, low density microparticles for inhalation are required to have geometric diameters between 5 and 30 μm in order to have suitable aerodynamic diameters^33^. The size analysis using laser diffraction confirmed that the average geometrical sizes of spray-freeze-dried silk-cisplatin and silk-cisplatin (cross-linked) particles were 22.75 μm and 10.08 μm, respectively (Fig. 2(a); (i,ii)). The cross-linking process demonstrated a general reduction in the overall particles. However these particles remained larger than spray-dried particles. As observed in SEM images, the spray-freeze-dried particles for both cross-linked and normal silk-based particles had higher porosity, which confirms their suitability for aerosolisation and lung deposition.

The inclusion of sugar in the spray-freeze-dried formulations was for their lyoprotectant properties. However, sugar has additional benefits such as the stability improvement and the prevention of agglomeration upon drying, as it acts as a matrix-former^34^. Initially trehalose or mannitol was included as lyoprotectants, but the formulations with trehalose were ruled out due to their inferior dispersibility in the *in vitro* aerosolisation, and therefore low lung deposition. After spray-freeze-drying the particles containing trehalose, SEM observations revealed that the particles were aggregated, the laser diffraction particle size distribution was wider and therefore it was inaccurate to report average particle sizes (Fig. 2(a); (iii)). The use of trehalose was also associated with increased hygroscopicity. Therefore, mannitol was found to be a superior lyoprotectant in the silk-based formulations.

#### Structure of particles and determination of crystallinity

The x-ray diffraction showed that the spray-dried Bm fibroin were amorphous as no peaks were present in the diffractogram (Fig. 2(b); (i)). When mannitol was used as the lyoprotectant, sharp peaks were present in the x-ray diffractogram of the formulations that represented crystalline structures (Fig. 2(b); (ii,iii)). The intensity of diffracted x-ray was slightly lower at around 40 degrees (2θ) in (iii) compared to (ii) and it can be seen that the cross-linking of silk fibroin with genipin led to a slightly less crystalline formulation compared to the non-cross-linked, spray-freeze-dried silk fibroin. However, there were minor differences overall and thus the effect of cross-linking with genipin was observed to have minimal impact on the level of crystallinity as well as the structure of the particles.

#### Particle dispersibility and *in vitro* testing for deposition in the lungs

The spray-dried particles were dispersed with almost no particles retained in the capsule inside an Aeroliser^®^ inhaler device, after it was subject to airflow of 60 L/min for the duration of 4 seconds. The fine particle fraction (FPF), the proportion of particles with aerodynamic diameter less than 5 μm, was calculated to be 61.67% (Fig. 2(c)).

The aerodynamic diameters of the particles were used by calculating the percentage of particles in each of the seven stages in the NGI. The stages on the NGI were separated by the cut-off diameters as stated in the figure legend of Fig. 2. The majority of the particles were deposited in stages 2 to 4, representing the particles with aerodynamic diameters between 8.06 and 2.82 μm at the airflow of 60 L/min (Fig. 2(d))^26^. A similar pattern was observed in the spray-freeze-dried Bm fibroin and FPF was calculated to be 62.25%, also similar to the spray-dried particles (data not presented). Although there were clear differences in the average geometric particle size according to the method of formulation, FPF and thus the aerosolisation efficiency were similar due to their porosity and low density, as per equation presented above.

These results showed that silk-based particles had high efficiency of aerosolisation, comparable or higher than those of dry powder inhaler formulations currently on the market, including Seretide^TM^ and Symbicort^TM^^35^,^36^,^37^. On the other hand, the FPF for spray-freeze-dried Bm fibroin with trehalose was unable to be determined as the majority of the particles were retained inside the capsule and could not be dispersed for *in vitro* testing.

### *In vitro* release of cisplatin

**Figure 3:**
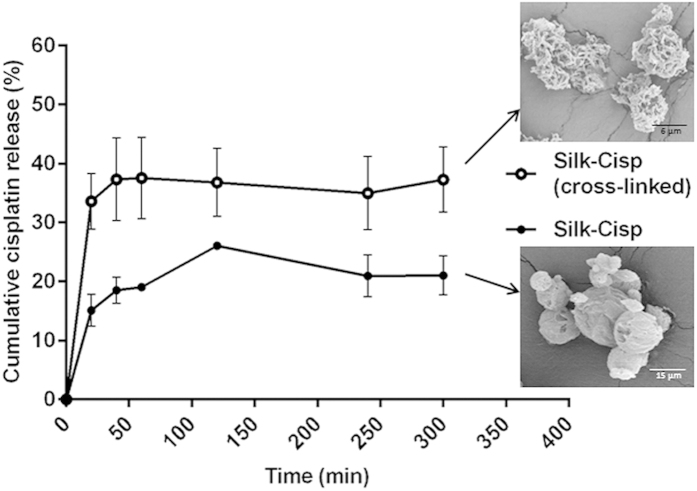
Cumulative percentage of cisplatin release in PBS (pH 7.4) from the silk-cisplatin formulations (with or without cross-linking) at 37 °C. The corresponding SEM images of the particles show morphological differences between the two formulations: Silk-Cisp (cross-linked silk; *above*) and Silk-Cisp (normal, non-cross-linked silk; *below*).

By engineering the formulation of silk-based particles, it was possible to regulate the extent of the cisplatin release in an *in vitro* model. The silk-cisplatin (cross-linked) particles produced a higher percentage of cisplatin (~40%) to be released in the first hour and then maintained a stable concentration thereafter (Fig. 3). The cisplatin release from non-cross-linked silk-cisplatin particles released ~18% of cisplatin in the first hour, followed by a small increase of drug released in the next points (Fig. 3).

The higher percentage release of cisplatin in the cross-linked formulation may have been due to the faster diffusion of cisplatin from the particles, which were highly porous (Fig. 3, SEM image insert). This morphology results in much greater surface contact area with the fluid and thus significantly increases the interactions with media and disintegration/degradation of the particles. In contrast, non-cross-linked particles were spherical, relatively smooth and characterized with compact structure. Hence, slower drug release could be related to smaller contact area of these particles with media and slow degradation process of the silk-based particles.

The potential benefits of faster diffusion and retaining the higher concentration of drug observed in the cross-linked formulation include the extended duration of action of drug and potentially improved patient compliance. Another finding was that mannitol included in the formulations as a lyoprotectant actually had additional benefits in enhancing drug release due to its osmotic characteristics^38^, assisting the cisplatin release from the silk fibroin which has slow degradation rate^39^.

### *In vitro* assays using silk-based particles to confirm cytocompatibility and cytotoxicity

#### Cell viability assays of silk-based particles

**Figure 4:**
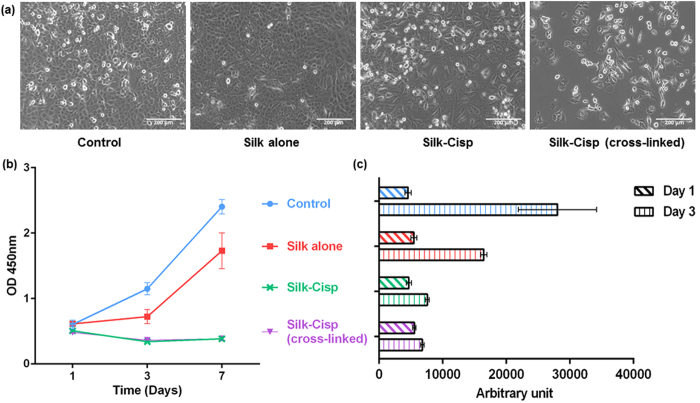
Comparison of cell viability and vitality in response to silk alone and silk-cisplatin particles (where silk is cross-linked or normal, as indicated); the concentration of particles used is 1 mg/ml (w/v) for all samples. (**a**) Phase microscopy showing differences in cell morphology depending on treatment. (**b**) The cell viability using CCK-8 assay: cell proliferation measured on days 1, 3 and 7 after treatment. (**c**) Quantification of double stranded DNA obtained from cells 1 and 3 days after treatment.

The morphology of the cells growing in the presence of silk alone was similar to cells in the control group that received no treatment with a slightly lower confluence. Distinct differences were observed in the cells treated with cisplatin-containing formulations compared to the control. There were also differences between the cross-linked and the normal silk (Fig. 4(a)).

Based on the CCK-8 assay silk fibroin was found to be cytocompatible. In the cells treated with silk alone the cell viability was comparable to the control only until day 1. Later, the pattern of the cell proliferation remained similar. The rate of cell proliferation for silk alone group was slower than control group (Fig. 4(b)). One-way ANOVA statistical analysis showed that the cell proliferation for control group was significantly higher than silk alone treatment group on day 7 only (p-values for day 1, 3 and 7 were 0.836, 0.056 and 0.017 respectively). These results correspond to the images in Fig. 4(a), as it can be seen that the human lung epithelial cells can proliferate normally in the presence of silk fibroin. The cisplatin-containing formulations with cross-linked or normal silk showed statistically significant suppression of growth of the cells from day 3 compared to control (p-values 0.021 and 0.019 for cross-linked and normal silk, respectively) (Fig. 4(b)). This cytotoxic effect was maintained until day 7 and was considered that the cisplatin was effectively carried and delivered to the cancer cells by the silk-based particles. Since the CCK-8 assay is a measure of metabolic activity of cells, it was necessary to measure the dsDNA content for more accurate estimate of the number of cells.

#### Double stranded DNA content (Picogreen^®^ assay)

The results from the cell viability assays were confirmed by dsDNA content assay using the Picogreen^®^ assay. The dsDNA content was comparable across all conditions on the first day after treatment. Clear differences were observed after 3 days of incubation (Fig. 4(c)). The number of cells in the samples treated with silk alone was less but comparable to the control group, which was in line with the results from the CCK-8 cell viability assay and the phase images. The cells treated with cisplatin had significant yet comparably minimal changes in dsDNA content from day 1 to day 3. Overall, these results were consistent with the results from the cell viability assay and supported that the metabolic activity of the cells correlated with the quantity of dsDNAs present. One important finding was that the metabolic activity measured in the cell viability assay also encompassed the increased metabolic activity of senescence cells. The smaller increase in the dsDNA content from day 1 to day 3 in the silk-cisplatin (cross-linked) formulation compared to the normal silk-cisplatin formulation was not apparent in the cell viability graph (Fig. 4(b,c)). These observations correspond well with our drug release study as discussed above. This suggests that the higher cytotoxicity of silk-cisplatin (cross-linked) formulation was due to higher extent of cisplatin release and the concentration of cisplatin maintained.

#### Cell metabolism

**Figure 5:**
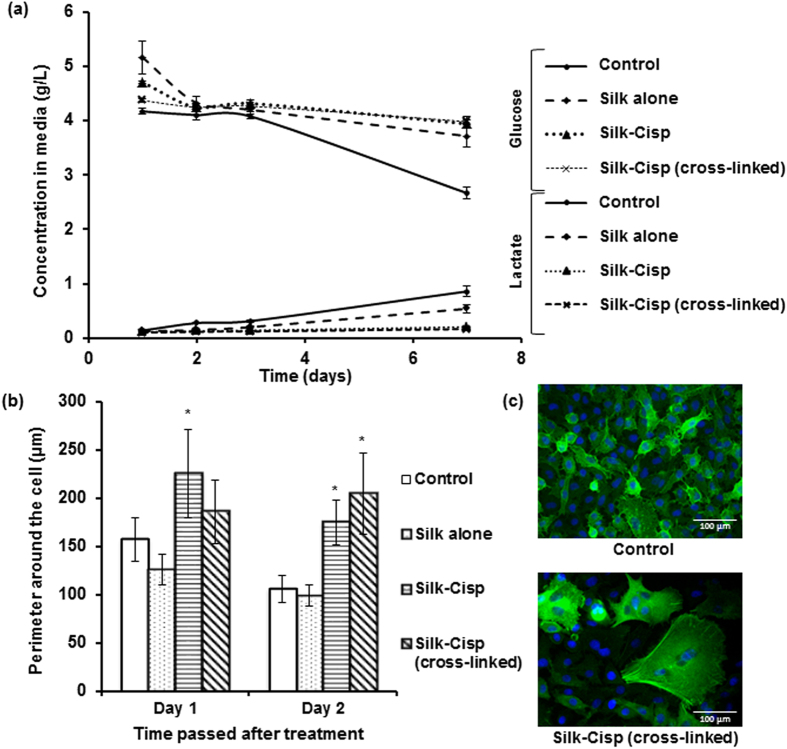
Comparison of the effects of various treatments on: (**a**) glucose and lactate levels detected in media in response to cell metabolism; (**b**) average size of cells at 1 and 2 days after treatment (*indicates statistically significant difference in cell size with the control group). (**c**) Immunofluorescence images after 2 days of treatment with normal media as control *(above)* and silk-cisplatin released from cross-linked silk *(below)*, stained with F-actin (green) and DAPI (blue).

The glucose concentration decreased by 1.5 g/L in the culture medium for control (no treatment), after seven days (Fig. 5(a)). The treatment with silk alone showed similar results with glucose concentration changed by 1.45 g/L after seven days of experiment. There were minor changes in the glucose concentration for the medium with cells treated with cisplatin delivered via normal or cross-linked silk, decreasing only by 0.76 g/L and 0.39 g/L respectively, after seven days. This reflected that there were certainly less number of cells that were metabolically active. They thus had subsequently reduced glucose uptake from the media, which is in agreement with the results from the CCK-8 cell viability assay. Similarly there was no significant lactate release over the seven days for cells treated with both normal and cross-linked silk-based formulations (Fig. 5(a)). This finding also supported the data from cell viability assays, as lactate is produced by mammalian cells as a metabolite by-product during the process while glucose is utilized for energy production^40^. The increases in lactate concentration in the media for the cells treated with silk alone were similar to the increase observed in the control group, which represented cell proliferation over time.

#### Changes in cell sizes and morphology

The surviving cells were characterised by varying sizes dependent on the formulation. The cells that received no treatment (control) had average perimeters of 106.68 μm whereas the cisplatin-containing normal and cross-linked silk-based particles led to cells having average perimeters of 175.24 μm and 205.15 μm respectively, after 48 hours of treatment with conditioned media (Fig. 5(b)). One-way ANOVA statistical analysis showed that the sizes of the cells treated with cisplatin released from non-cross-linked silk were significantly larger compared to control at both day 1 and day 2 (p-values 0.004 and 0.014 respectively). The cross-linked silk had significant effect on cell size only at day 2 and not day 1 (Fig. 5(b)) (p-value for day 2 was 0.001). The enlargement of cells was indicative of senescence-like cells, where they were metabolically active, but failed to divide upon being treated with cisplatin^41^.

The effect of cisplatin released from the cross-linked silk formulation was remarkably evident in the reduced number of cells. The change in the shape of surviving cells and the presence of senescence-like cells were observed along with the enlarged actin (Fig. 5(c)). Furthermore, considerably less number of nuclei (shown in blue; stained with DAPI) were present after 2 days of treatment with cisplatin compared to the control, which represented less number of live cells.

#### Wound repopulation assay

**Figure 6:**
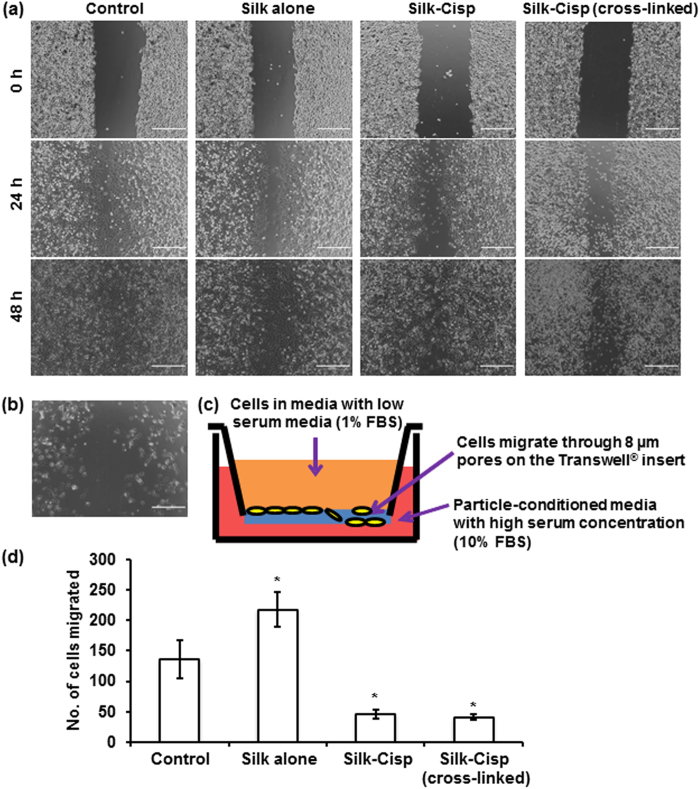
(**a**) Phase images of the cells in wound repopulation assay. The “wound” closed for the control group and the cells treated with silk alone within 48 hours. The “wound” gap remains unclosed for the cells treated with silk-cisplatin formulations regardless of the formulation type. (**b**) A phase image of the cells treated with silk-cisplatin (cross-linked) formulation for 48 hours, after washing with PBS – only small number of viable cells was observed; the “wound” gap did not close. (**c**) A schematic diagram of the cell migration experimental setup. (**d**) Comparison of the number of cells migrated in “suppressed attraction” assay (*indicates statistically significant difference in the number of cells migrated compared with the control group). Scale bars represent 400 μm.

After 2 days of treatment, the areas of the wounds were almost completely closed by the cells treated with normal media and media containing silk alone. In contrast, the wounds remained unclosed for those treated with cisplatin. The cells treated with formulations with silk alone or silk with sugar had repopulated and closed the wound in a similar manner to control (Fig. 6(a)). The cells treated with cisplatin-containing normal or cross-linked silk formulations did not repopulate. The cytotoxic treatment had detached and killed the cells that were attached on the tissue culture plate (Fig. 6(b)).

#### Cell migration

The suppressed movement of the cells in response to the particles was monitored using the presence of the FBS concentration gradient from 1% in the upper chamber and 10% in the lower chamber (Fig. 6(c)). The cells were found to move favourably from the upper chamber to the lower chamber containing silk alone and significantly less number of cells migrated when cisplatin-containing particles were present in the lower chamber (Fig. 6(d)). There were no significant differences observed in the migration of cells treated with cisplatin delivered via cross-linked or normal silk. The differences in cell responses between these two samples may be greater if the formulation is modified, possibly by using a higher concentration of genipin or by altering the cross-linking time to achieve a more extensively cross-linked silk fibroin.

## Conclusion

Inhalable silk particles are successfully formulated to act as carriers of the drug for the targeted delivery to the lungs. The aerosolisation performance of the particles demonstrates the ability for adequate dispersion and reaching down to the lower airways. Cisplatin incorporated into silk-based carriers demonstrate the potential to deliver cisplatin directly to the lungs via dry powder inhalers instead of the conventional injection method. The optimized formulations of silk-based drug carriers are shown to be cytocompatible with A549 lung epithelial cell line. The cells show normal growth and proliferation in the presence of silk protein in the media. Silk fibroin is cross-linked with genipin for a modified release of cisplatin. Cytotoxicity investigation showed that the cross-linked formulation released cisplatin more effectively. Thus it had more potent cytotoxic effect compared to the normal silk-cisplatin formulation. Future investigation may be carried out with the optimisation of the formulations to achieve desired release rates, for instance by combining the normal and cross-linked silk-based formulations. This also could lead to further exploration of the release mechanism of drug from silk-based formulations. Our fabricated silk-based delivery system may lead to a more effective targeted delivery of cisplatin to the lung cancer cells.

## Additional Information

**How to cite this article**: Kim, S. Y. *et al.* Formulation of Biologically-Inspired Silk-Based Drug Carriers for Pulmonary Delivery Targeted for Lung Cancer. *Sci. Rep.* **5**, 11878; doi: 10.1038/srep11878 (2015).
